# Supplementary material for: Burden of diabetic ketoacidosis and its predictors among diabetic patients in Ethiopia: Systematic review and meta-analysis
Source: PLoS One. 2025 Jan 23;20(1):e0309097. doi: 10.1371/journal.pone.0309097 (PMC11756790; doi:10.1371/journal.pone.0309097)
Supplement: S1 Table — (DOCX) [file pone.0309097.s001.docx]

| Studies | **Selection** | | | | **Comparability** | **Outcome** | | **Total** | **Risk of bias** |
| --- | --- | --- | --- | --- | --- | --- | --- | --- | --- |
|  | Representativeness of the sample | Sample size | Non-respondents | Ascertainment of the screening/surveillance |  |  |  |  |  |
|  |  |  |  |  |  | Assessment of outcome | Statistical test |  |  |
| 1. Assefa et al | * | * | * | * | * | ** | * | ******** | Low |
| 1. Asrat et al | * | * | * | * | * | * | * | ******* | Low |
| 1. Debela et al | * | * |  | * | * | * | * | ****** | Moderate |
| 1. Tigstu et al | * | * | * | ** | * | * | * | ******** | Low |
| 1. Eskeziya et al | * | * | * | * | * | * | * | ******* | Low |
| 1. Gebre et al | * | * | * | * | * | ** | * | ******** | Low |
| 1. Fikaden et al | * | * | * | * | * | * | * | ******* | Low |
| 1. Negera et al | * | * |  | * | * | * | * | ****** | Moderate |
| 1. Bedaso et al | * | * | * | * | * | * | * | ******* | Low |
| 1. mengistu et al | * | * | * | * | * | * | * | ******* | Low |
| 1. Zeleke et al | * | * |  | * | * | ** | * | ******* | Low |
| 1. Leblo et al | * | * | * | * | * | * | * | ******* | Low |
| 1. Tekeste et al | * | * | * | * | * | * | * | ****** | Low |
| 1. Gedamu et al | * | * | * | * | * | * | * | ******* | Low |
| 1. Kefale et al | * | * | * | * | * | * | * | ******* | Low |
| 1. Abate et al | * | * | * | * | * | * | * | ******* | Low |
| 1. Desse et al | * | * |  | * | * | * | * | ****** | Moderate |
| 1. Eyob et al | * | * | * | * | * | * | * | ******* | Low |
| 1. Tola et al | * | * | * | * | * | * | * | ******* | Low |
| 1. Korsa et al | * | * | * | * | * | * | * | ******* | Low |
| 1. Tilaye et al | * | * |  | ** | * | * | * | ****** | Low |
| 1. Gizaw et al | * | * | * | * | * | * | * | ******* | Low |
| 1. Kidie et al | * | * | * | * | * | * | * | ******* | Low |

***The three main categories in the NOS are: Selection, Comparability and Outcomes with some further subdivisions.***

***Selection: representative cases (*), justified sample size (*), satisfactory response rate (*), validated tools (2*).***

***-Comparability: comparable groups and confounding factors are controlled (*).***

***-Outcome: independent blind assessment (2*), record linkage (2*), self-report (*), appropriate statistical test (*).***

***Then we classify low bias ≥7 points, moderate bias 3-6 points, high bias ≤3points***
