# Supplementary material for: Burden of diabetic ketoacidosis and its predictors among diabetic patients in Ethiopia: Systematic review and meta-analysis
Source: PLoS One. 2025 Jan 23;20(1):e0309097. doi: 10.1371/journal.pone.0309097 (PMC11756790; doi:10.1371/journal.pone.0309097)
Supplement: S2 Table — (DOCX) [file pone.0309097.s002.docx]

| **S.no** | **Authors** | **Full title** | **Year** | **Included (yes/no)** | **If no, reason for exclusions*** |
| --- | --- | --- | --- | --- | --- |
|  | Assefa et al | Incidence and predictors of diabetic ketoacidosis among children with diabetes in west and east Gojjam zone referral hospitals, northern Ethiopia, 2019 | 2020 | Yes |  |
|  | Asrat et al | Diabetic Complications among Adult Diabetic Patients of a Tertiary Hospital in Northeast Ethiopia | 2015 | Yes |  |
|  | Debela et al | Characteristics and burden of diabetic ketoacidosis in diabetic patients in the period of COVID-19 outbreak in Ethiopia | 2023 | Yes |  |
|  | Tigstu et al | Predictors and treatment outcome of hyperglycemic emergencies at Jimma University Specialized Hospital, southwest Ethiopia | 2015 | Yes |  |
|  | Eskeziya et al | Prevalence of Diabetic Keto Acidosis and Associated Factors among Newly Diagnosed Patients with Type One Diabetic Mellitus at Dilla University Referral Hospital, September 9th/2017 – May 30th/2019: South Ethiopia; Crossectional Study | 2020 | Yes |  |
|  | Gebre et al | Magnitude and associated factors of diabetic complication among diabetic patients attending Gurage zone hospitals, South West Ethiopia | 2019 | Yes |  |
|  | Fikaden et al | Diabetic ketoacidosis in children and adolescents with newly diagnosed type 1 diabetes in Tigray, Ethiopia: retrospective observational study | 2019 | Yes |  |
|  | Negera et al | Acute Complications of Diabetes and its Predictors among Adult Diabetic Patients at Jimma Medical Center, Southwest Ethiopia | 2020 | Yes |  |
|  | Bedaso et al | Diabetic ketoacidosis among adult patients with diabetes mellitus admitted to emergency unit of Hawassa university comprehensive specialized hospital | 2019 | Yes |  |
|  | mengistu et al | OCCURRENCE AND FACTORS ASSOCIATED WITH DIABETIC KETOACIDOSIS AMONG CHILDREN SEEN AT HAWASSA UNIVERSITY, COMPREHENSIVE PECIALIZED HOSPITAL: A CROSS-SECTIONAL STUDY | 2020 | Yes |  |
|  | Zeleke et al | Incidence and predictors of diabetic ketoacidosis among children with diabetes in west and east Gojjam zone referral hospitals, northern Ethiopia, 2019 | 2019 | Yes |  |
|  | Leblo et al | Diabetic Ketoacidosis Among Type-I Diabetes Patients Who Visited Menelik-Ii Referral Hospital in Addis Ababa, 2022 | 2023 | Yes |  |
|  | Tekeste et al | Treatment Outcome of Hyperglycemic Emergency and Predictors in Ethiopia | 2020 | Yes |  |
|  | Gedamu et al | Diabetic Complications and Associated Factors among Diabetic Patients in Tikur Anbessa Specialized Hospital, Addis Ababa, Ethiopia | 2022 | Yes |  |
|  | Kefale et al | Hospitalization Pattern and Treatment Outcome Among Diabetic Patients Admitted to a Teaching Hospital in Ethiopia: A Prospective Observational Study | 2016 | Yes |  |
|  | Abate et al | Incidence and predictors of hyperglycemic emergencies among adult diabetic patients in Bahir Dar city public hospitals, Northwest Ethiopia, 2021: A multicenter retrospective follow-up study | 2023 | Yes |  |
|  | Getie et al | Determinants of diabetes ketoacidosis among diabetes mellitus patients at North Wollo and Wagehemira zone public hospitals, Amhara region, Northern Ethiopia | 2015 | Yes |  |
|  | Eyob et al | In-Hospital Mortality and Its Predictors among Hospitalized Diabetes Patients: A Prospective Observational Study | 2023 | Yes |  |
|  | Tola et al | Magnitude and predictors of hospital admission, readmission, and length of stay among patients with type 2 diabetes at public hospitals of Eastern Ethiopia: a retrospective cohort study | 2021 | Yes |  |
|  | Korsa et al | Diabetes Mellitus Complications and Associated Factors Among Adult Diabetic Patients in Selected Hospitals of West Ethiopia | 2019 | Yes |  |
|  | Tilaye et al | Assessment of Acute Complications of Diabetes Mellitus Using Clinical Records of Diabetic Patients in Adama Hospital Medical College, Oromia Regional State, Ethiopia | 2021 | Yes |  |
|  | Gizaw et al | Diabetes mellitus in Addis Ababa, Ethiopia: admissions, complications and outcomes in a large referral hospital | 2015 | Yes |  |
|  | Kidie et al | Frequency of Diabetic Ketoacidosis and Its Determinants Among Pediatric Diabetes Mellitus Patients in Northwest Ethiopia | 2021 | Yes |  |
|  | Atkilt1 et al | Clinical Characteristics of Diabetic Ketoacidosis in Children with Newly Diagnosed Type 1 Diabetes in Addis Ababa, Ethiopia: A Cross-Sectional Study | 2017 | No | Outcome of interest not explained |
|  | Bekele et al | Prevalence and Associated Factors of Carpal Tunnel Syndrome Among Diabetic Patients in Arba Minch General Hospital, South West Ethiopia, 2021 | 2022 | No | Outcome of interest not explained |
|  | Derse et al | Outcome of Diabetic Keto Acidosis Treatment and Associated Factors Among Adult Patients Admitted to Emergency and Medical Wards at St. Paul’s Hospital, Addis Ababa Ethiopia, 2023: A Cross-Sectional Study | 2023 | No | Outcome of interest not explained |
|  | Ebrahim et al | Prevalence and associated factors of depression among diabetic outpatients attending diabetic clinic at public hospitals in Eastern Ethiopia: A cross-sectional study | 2021 | No | Outcome of interest not explained |
|  | Ejeta et al | Diabetes Concordant Comorbidities and Associated Factors Among Adult Diabetic Out-Patients at Hiwot Fana Specialized University Hospital, Harar, Eastern Ethiopia: A Cross-Sectional Study | 2021 | No | Outcome of interest not explained |
|  | Meseret et al | Treatment Outcome and Associated Factors among Type 1 Diabetic Children Admitted with DKA in Bahir Dar City Public Referral Hospital, Northwest, Ethiopia: A Cross-sectional Study. | 2024 | No | Outcome of interest not explained |
|  | Shibeshi et al | Pediatric diabetic retinopathy: experience of a tertiary hospital in Ethiopia | 2016 | No | Out of the scope |
|  | Shiferaw et al | Diabetic ketoacidosis (DKA) induced cerebral edema complicating small chronic subdural hematoma/hygroma/ at Zewuditu memorial hospital: a case report | 2017 | No | Outcome of interest not explained |
|  | Shimelash et al | Incidence and predictors of mortality in children with diabetic ketoacidosis in the comprehensive specialized referral hospitals of West Amhara Region, Northwest Ethiopia: a retrospective follow-up study. | 2023 | No | Excluded after reading the full text |
|  | Shumye et al | Prevalence and associated factors of proliferative diabetic retinopathy among adult diabetic patients in Northwest Ethiopia, 2023: A cross-sectional multicenter study | 2024 | No | Excluded after reading the full text |
|  | Abegaz et al | Treatment outcome of diabetic ketoacidosis among patients attending general hospital in north-West Ethiopia: hospital-based study | 2018 | No | Excluded after reading the full text |
|  | Alemnew et al | Clinical characteristics, precipitating factors and glycemic control among diabetic ketoacidosis patients admitted to university hopsital in Northwest Ethiopia: A hospital based observational study | 2019 | No | Out of scope |
|  | Asmelash et al | Knowledge, attitude, and practice towards glycemic control and its associated factors among diabetes mellitus patients | 2019 | No | Excluded after reading the full text |
|  | Bacha et al | Outcome of diabetic ketoacidosis among paediatric patients managed with modified DKA protocol at Tikur Anbessa specialized hospital and Yekatit 12 hospital, Addis Ababa, Ethiopia | 2022 | No | Out of the scope |
|  | De Vries et al | Factors associated with diabetic ketoacidosis at onset of Type 1 diabetes in children and adolescents | 2013 | No | Out of scope |
|  | Dereje et al | Characteristics of diabetic ketoacidosis in adult patients in FH Jimma, Oromia, Ethiopia, 2022 | 2023 | No | Excluded after reading the full text |
|  | Tsega et al | Outcome of Diabetic Keto Acidosis Treatment and Associated Factors Among Adult Patients Admitted to Emergency and Medical, | 2020 | No | Out of scope |
|  | Zegeye et al | Prevalence and factors associated with Diabetes retinopathy among type 2 diabetic patients at Northwest Amhara Comprehensive Specialized Hospitals, Northwest Ethiopia 2021 | 2023 | No | Out of scope |
| 42. | Sheleme T et al | Prevalence, patterns and predictors of chronic complications of diabetes mellitus at a large referral hospital in Ethiopia: a prospective observational study. Diabetes, Metabolic Syndrome and Obesity. 2020 Dec 11:4909-18. | 2020 | No | Out of scope |
| 43. | Belsti Y et al | Awareness of complications of diabetes mellitus and its associated factors among type 2 diabetic patients at Addis Zemen District Hospital, northwest Ethiopia. BMC research notes. 2019 Dec; 12:1-7. | 2019 | No | Out of scope |
| 44. | Seid MA et al | Microvascular complications and its predictors among type 2 diabetes mellitus patients at Dessie town hospitals, Ethiopia. Diabetology & Metabolic Syndrome. 2021 Dec;13:1-8. | 2021 | No | Out of scope |
| 45 | Wolde HF et al | Predictors of vascular complications among type 2 diabetes mellitus patients at University of Gondar Referral Hospital: a retrospective follow-up study. BMC endocrine disorders. 2018 Dec; 18:1-8. | 2018 | No | Outcome of interest was not found |
| 46 | Kifle ZD et al | Knowledge towards diabetes and its chronic complications and associated factors among diabetes patients in University of Gondar comprehensive and specialized hospital, Gondar, Northwest Ethiopia. Clinical Epidemiology and Global Health. 2022 May 1;15:101033 | 2022 | No | Outcome of interest was not found |
| 47 | Tesfaye DJ | Coexistence of chronic complications among diabetic patients at nigist eleni mohammed memorial hospital, hossana, south Ethiopia | 2015 |  | Outcome of interest was not found |
| 48 | Shita NG et al | Predictors of blood glucose change and microvascular complications of type 2 diabetes mellitus patients in Felege Hiwot and Debre Markos referral hospital, North West Ethiopia. BMC Endocrine Disorders. 2022 May 23;22(1):136. | 2022 | No | Outcome of interest was not found |
| 49 | Chekol GZ et al | Is the Duration of Diabetes Diseases Positively Associated with Knowledge About Diabetic Complications? Knowledge of Diabetes Mellitus Complications and Associated Factors Among Type-2 Diabetic Patients in Public Hospitals of Addis Ababa, 2020. Frontiers in Public Health | 2020 | No | Outcome of interest was not found |
| 50 |  |  |  |  |  |
| 51 | Yeheyis et al | The Pattern of Initial Presentation of Diabetes, Treatment Outcome and Its Predictors Among Diabetic Pediatrics Attended Service at Selected Public Hospitals of Southern Ethiopia: A Multi-Center Study | 2023 | No | Out of scope |
| 52 | Tilahun et al | Prevalence of Diabetic Retinopathy and Its Associated Factors among Diabetic Patients at Debre Markos Referral Hospital, Northwest Ethiopia, 2019: Hospital-Based Cross-Sectional Study. | 2020 | No | Out of scope |
| 53 | Tekeste et al | Treatment outcome of hyperglycemic emergency and predictors in Ethiopia. | 2020 | No | Excluded after reading the full text |
| 54 | Taye et al | Diabetic ketoacidosis management and treatment outcome at medical ward of Shashemene Referral Hospital, Ethiopia: a retrospective study | 2021 | No | Excluded after reading the full text |
| 55 | Muluwork et al | Assessment of Clinical Profile, And Treatment Outcome of Diabetic Ketoacidosis among Diabetic Children | 2020 | No | Excluded after reading the full text |
| 56 | Mekonnen et al | Treatment outcomes of diabetic ketoacidosis among diabetes patients in Ethiopia. Hospital-based study. | 2022 | No | Excluded after reading the full text |
| 57 | Mariam et al | Prevalence of Diabetic Foot Ulcer and Associated Factors among Adult Diabetic Patients Who Attend the Diabetic Follow-Up Clinic at the University of Gondar Referral Hospital, North West Ethiopia, 2016: Institutional-Based Cross-Sectional Study | 2017 | No | Out of scope |
| 58 | Kidie et al | Poor glycemic control and associated factors among pediatric diabetes mellitus patients in northwest Ethiopia, 2020: facility-based cross-sectional retrospective study design | 2022 | No | Out of scope |
| 59 | Kefale et al | Hospitalization pattern and treatment outcome among diabetic patients admitted to a teaching Hospital in Ethiopia: a prospective observational study | 2016 | No | Out of scope |
| 60. | Eshetu et al | Assessment of Electrolyte Imbalance and Associated Factors Among Adult Diabetic Patients Attending the University of Gondar Comprehensive Specialized Hospital, Ethiopia: A Comparative Cross-Sectional Study | 2023 | No | Out of scope |

* The number of articles included and excluded (with reasons for exclusion clearly detailed in the PRISMA flow diagram)

Missing data were managed by using complete case analysis
