# Supplementary material for: Burden of diabetic ketoacidosis and its predictors among diabetic patients in Ethiopia: Systematic review and meta-analysis
Source: PLoS One. 2025 Jan 23;20(1):e0309097. doi: 10.1371/journal.pone.0309097 (PMC11756790; doi:10.1371/journal.pone.0309097)
Supplement: S1 Annex — (DOCX) [file pone.0309097.s005.docx]

**Annex**

**Annex I**

**MeSH terms and databases.** We employed specific terms to search for primary studies, using phrases like "magnitude" OR "prevalence" in conjunction with "diabetic ketoacidosis" AND “diabetes patients" in the context of Ethiopia. To explore factors linked with diabetic ketoacidosis, we utilized terms such as "factors," "determinants," "risk factors," "correlations," OR "predictors" alongside "diabetic ketoacidosis. Database used for article retrieval for burden and predictors of DKA among DM patients in Ethiopia as shown in (**Table 1**).

**Table 1. Database used for article retrieval for burden and predictors of DKA among DM patients in Ethiopia**

| **Data bases** | **Number of article retrieved** |
| --- | --- |
| Google scholar | 112 |
| PubMade | 29 |
| Science direct | 42 |
| Gray literatures | 6 |

**Annex II**

**Table 2. Subgroup analysis of pooled prevalence of DKA among DM patients by study participant and area in Ethiopia.**

|  | **Subgroups** | **Number of studies (K)** | **Proportion (95 %CI )** |
| --- | --- | --- | --- |
| Study participant | Children | 4 | 68.2% (59.32%, 77.10%) |
|  | Both Adult and Children | 5 | 57.78% (38.10, 77.50%) |
|  | Adult | 13 | 35.45% (21.67%, 49.23) |
| Study area | Amhara | 4 | 52. 44% (35.76%, 69.12% ) |
|  | Oromia | 8 | 49.83% (26.03%, 73.63%) |
|  | Southern | 4 | 44.95% (26.91%, 62.99%) |
|  | Tigray | 1 | 78.66% (74.22%, 83.09%) |
|  | Addis Ababa | 4 | 32. 52% (12.05%, 52.99%) |
|  | Harari | 1 | 24.02% (20.11%, 27.93%) |
