# Supplementary material for: Burden of diabetic ketoacidosis and its predictors among diabetic patients in Ethiopia: Systematic review and meta-analysis
Source: PLoS One. 2025 Jan 23;20(1):e0309097. doi: 10.1371/journal.pone.0309097 (PMC11756790; doi:10.1371/journal.pone.0309097)
Supplement: Attachment — Submitted filename: Editors comment.docx [file pone.0309097.s006.docx]

| Editors comment | Author response |
| --- | --- |
| Please ensure that your manuscript meets PLOS ONE's style requirements, including those for file naming. | Thank you very much for insight. We made a revision on it. |
| We note that your Data Availability Statement is currently as follows: All relevant data are within the manuscript and its Supporting Information files  Please confirm at this time whether or not your submission contains all raw data required to replicate the results of your study. Authors must share the “minimal data set” for their submission. PLOS defines the minimal data set to consist of the data required to replicate all study findings reported in the article, as well as related metadata and methods (<https://journals.plos.org/plosone/s/data-availability#loc-minimal-data-set-definition>). | Thank you very much for insight. We made a revision on it. |
| Please ensure that you refer to Figure 2, 5 and 6 in your text as, if accepted, production will need this reference to link the reader to the figure. | Thank you very much for insight. We made a revision on it. |
| Please include captions for your Supporting Information files at the end of your manuscript, and update any in-text citations to match accordingly. Please see our Supporting Information guidelines for more information: <http://journals.plos.org/plosone/s/supporting-information>. | Thank you very much for insight. We made a revision on it. |
| There should be evidence of registration on PROSPERO before we progress further with this manuscript. | Thank you very much for insight. We made a revision on it. |
| **Reviewer one** |  |
| I suggest placing all MeSH terms in an appendix instead of listing them within the main manuscript. Additionally, provide a table format listing all the searching databases along with their respective numbers of articles found. It's worth noting that the current searching databases may not be sufficient to capture all relevant literature, potentially leading to missed papers due to inadequate search strategies. | Thank you very much for insight. We made a revision on it. |
| In terms of eligibility criteria, could you clarify whether the authors excluded studies with duplicate published literature and those lacking essential outcome data? This clarification is crucial to address potential duplication. Additionally, you've listed observational studies reporting the prevalence of diabetic ketoacidosis in individuals with diabetes patients in Ethiopia between 2013 and 2023. However, could you justify the exclusion of the following articles: Getie 2021, Asfaw 2021, Eyob Tediso 2023, Korsa 2019, Tilaye 2021, Abate 2023, Kefale 2016, Desse 2015, Gizaw 2015, and Tola 2021? | Thank you very much for insight. We made a revision on it. |
| In the Outcome Measurements section, please elaborate on the primary and secondary outcomes of the study. | Thank you for your insightful comments.  We made a revision. |
| In the Statistical Analysis section, considering you reported associations using adjusted odds ratios, it is recommended to report the extraction of AOR with their upper and lower bounds in the analysis section. Could you also provide the I2 ranges to assess heterogeneity and include a reference? Subgroup analysis should include the type of diabetes mellitus as it is crucial for diabetic ketoacidosis prevalence. Additionally, please mention the performance of sensitivity analysis to examine the influence of a single study on the overall estimate under the analysis section. | Thank you very much for insight. We made a revision on it. |
| In the Results section: • Keep the same searching databases: Google Scholar, PubMed/Medline, Web of Sciences, and grey literature. • Regarding Figure 1, where "53 articles were excluded," include the issue of duplication under the eligibility criteria. • The overall number of participants should be provided when assessing the pooled prevalence (e.g., From 14 studies with ‘__’ study participants, the pooled prevalence of DKA among DM patients in Ethiopia was…). | Thank you very much for insight. We made a revision on it. |
| The Discussion should commence by highlighting the main findings (the pooled prevalence and associated factors) before discussing them separately. It would be beneficial if the authors compared the findings with those from high-income countries for coherence." | Thank you very much for insight. We made a revision on it. |
| **Reviewer 2** |  |
| Rewrite the methods section of the abstract. | Thank you very much for your insightful comments  We made a revision. |
| There is continuous repetition of ideas in the last paragraph-h of the introduction please reexamine and correct it. | We acknowledge your insightful comment. We made a revision on it. |
| In you document you used the phrase “diabetic patients instead of “diabetic patients”. | We acknowledge your insightful comment. We made a revision on it. |
| There are also many typos errors through the manuscript or to be rephrase paragraphs please wisely address it. | We acknowledge your insightful comment. We made a revision on it. |
| In the methods, you should have complied with the PRISMA (Preferred Reporting Items for Systematic Reviews and Meta-analyses) guidelines. Why did you strictly follow the PRISMA 2020 Checklist and PRISMA 2020 flow diagram? Since figure 1 is not similar to the PRISMA 2020 flow diagram, please visit this link: PRISMA ([prisma-statement.org](http://prisma-statement.org/)) | Thank you very much for insight. We made a revision on it. |
| Please check the sensitivity analysis (the impact of each study on the pooled effect size). | Thank you very much for insight. We made a revision on it. |
| State any a priori levels of significance and whether the tests were 1- or 2-sided. | Thank you very much for insight. We made a revision on it. |
| Some of your discussions are ambitious; as such, they are not supported by sufficient research or study across the globe. It should be solved for the resubmitted or revised manuscript. | Thank you very much for insight. We made a revision on it. |
| Where are other statistical results like Begg’s test? | Thank you.  For assessing publication bias, **Egger's test** is generally considered better due to its higher sensitivity. Or, Begg’s is less sensitive and may fail to detect publication bias, especially when the number of studies is small or when there is mild to moderate bias. |
| There is repetition of ideas throughout the manuscript. | We acknowledge your insightful comment. We made a revision on it. |
| Did you think you searched the available articles extensively? |  |
| All abbreviations you use in your manuscript should be written in full the first time, and you should use the abbreviated form in other parts of your manuscript. | We acknowledge your insightful comment. We made a revision on it. |
| The references are from 2003 to 2021. It is better to update the old references if there are any currently conducted articles available across the globe | Thank you very much for insight. We made a revision on it. |
| There are many more editorial, punctuation, and language problems. Please address these issues with English-language professionals. | Thank you very much for insight. We made a revision on it. |
| Finally, please check all references to ensure that none of the cited articles have been retracted. You can use the Retraction Watch database, available here (<http://retractiondatabase.org/>) | Thank you very much for insight. We made a revision on it. |
