# Supplementary material for: Burden of diabetic ketoacidosis and its predictors among diabetic patients in Ethiopia: Systematic review and meta-analysis
Source: PLoS One. 2025 Jan 23;20(1):e0309097. doi: 10.1371/journal.pone.0309097 (PMC11756790; doi:10.1371/journal.pone.0309097)
Supplement: Attachment — Submitted filename: Rebuttal letter.docx [file pone.0309097.s007.docx]

| **Reviewers comments** | **Authors response** |
| --- | --- |
| Reviewer 2 |  |
| 1. Dear authors, as far as I know one work found below is existed regarding DKA. Therefore, at the end paragraph of your introduction please state the gabs of the previously published work and state what you fill in current work. Abera, E. G., Yesho, D. H., Erega, F. T., Adulo, Z. A., Gebreselasse, M. Z., & Gebremichael, E. H. (2024). Burden of diabetic ketoacidosis among patients with diabetes mellitus in Ethiopia: a systematic review and meta-analysis. BMJ open, 14(2), e077151. <https://doi.org/10.1136/bmjopen-2023-077151> | Thank you very much for your insight full comments  We made a revision according to it. |
| 2. The databases mentioned in the abstract (PubMed/MEDLINE, Google Scholar, and gray literature) and method (Google Scholar, PubMed/Medline, Web of sciences, and grey literature) section varies. How it happen? | Thank you very much.  We made a revision on it. |
| 3. Please perform sensitivity analysis | Thank you for your insight  We made it. |
| 4. In the discussion section, your study did not give credit for African and Asian country studies. Also, your study result is not well discussed with the previous systematic review and meta-analysis studies conducted in Ethiopia and abroad. | Thanks for your constructive comments:  We made a revision on it. |
| 5. In your study, the justifications written in the discussion sections of all paragraphs are not supported by previously published/conducted studies. | We would like to acknowledge for your deep insight: We made a revision on it. |
| 6. Please check the language again. | We would like to acknowledge for your deep insight : We made a revision on it. |
| **Editor’s comment** |  |
| 1. Please review your reference list to ensure that it is complete and correct. If you have cited papers that have been retracted, please include the rationale for doing so in the manuscript text, or remove these references and replace them with relevant current references. Any changes to the reference list should be mentioned in the rebuttal letter that accompanies your revised manuscript. If you need to cite a retracted article, indicate the article’s retracted status in the References list and also include a citation and full reference for the retraction notice | We made a revision on it. |
